# Caspase Cleaved Tau in Alzheimer’s Disease: A Therapeutic Target Realized

**DOI:** 10.23937/2378-3001/2/1/1014

**Published:** 2015-01-15

**Authors:** Troy T. Rohn

**Affiliations:** Department of Biological Sciences, Boise State University, USA

**Keywords:** Tau, Caspase 3, neurofibrillary tangles, Alzheimer’s disease, cleavage

Alzheimer’s disease (AD) is a progressive neurodegenerative disorder characterized by an array of symptoms affecting memory and cognition. Some common symptoms of AD include memory loss that disrupts daily life, challenges in planning or solving problems, confusion with time or place, and changes in mood and personality [1]. Central dogma to the etiology of AD is the beta-amyloid cascade, which stipulates that beta-amyloid in oligomeric forms represents the earliest step in a cascade eventually leading to the formation of senile plaques and neurofibrillary tangles (NFTs) and neurodegeneration [2]. For many years the connection between plaques and tangles was unknown, however, in 2002 we reported that caspase activation and the cleavage of tau might link these two molecular entities in AD [3]. Our evidence was based on the synthesis and application of a caspase-cleavage site directed antibody to a known caspase-cleavage site within tau located at the amino-terminus (position 25). Figure 1 depicts the first experiment ever performed with affinity-purified tau caspase-cleavage antibody that revealed widespread labeling predominantly within NFTs, neuropil threads, and dystrophic neurites (Figure 1A) that was absent in age-matched control sections (Figure 1B).

The model we proposed in a subsequent review article was the activation of apoptotic pathways by beta-amyloid leads to the cleavage of tau and promotes the formation of NFTs in the AD brain [4]. Shortly thereafter, two studies largely confirmed our hypothesis by demonstrating that the caspase cleavage of tau is an early event in NFT evolution, and links beta-amyloid to NFT formation in the AD brain [5,6]. Both studies relied heavily on data obtained using identical site-directed antibodies to the C-terminal caspase-cleavage site within tau located at amino acid residue D421 and supported a general role for caspase-3 as being the major executioner protease involved in cleaving tau at this C-terminal site. The conclusion from both studies was that the caspase-cleavage of tau was an early event in AD disease tangle pathology and that caspase-3 may serve as the link between beta-amyloid deposition and the formation of NFTs [7]. Subsequently, several studies have confirmed a key role for caspase activation and the cleavage of tau as a proximal event in promoting tangle pathology [8–14]. Of great value to the field, the antibody developed by Lester Binder’s group at Northwestern University was made available commercially and this antibody, known as TauC3 has been instrumental in documenting the role of caspase-mediated truncation of tau in AD. By all accounts, this monoclonal antibody is an excellent antibody that shows no reactivity with full-length tau or other tau C-terminal truncations and is specific for NFTs, and caspase-cleaved tau within neuritic plaques and neuropil threads [12].

Based on the role of caspase-mediated cleavage of tau in promoting NFT formation in AD, blocking this cleavage event may provide a potential therapeutic strategy for the treatment of this disease. Recently Intellect Neurosciences, Inc. acquired the worldwide development and commercialization rights to TauC3 under an exclusive license agreement with Northwestern University. This past year, Intellect Neurosciences announced it obtained proof of concept in a preclinical Alzheimer’s model for its TauC3 monoclonal antibody indicating its potential utility as a therapeutic agent. The study was carried out in collaboration with University of California, Irvine’s Dr. Frank LaFerla, Director, Institute for Memory Impairments and Neurological Disorders as well as Dr. Kim Green. The results from this unpublished study showed that the TauC3 antibody “effectively engaged the target and reduced phosphorylated pathological forms of Tau indicating that the treatment with the peripherally administered antibody had an effect in the brain and is able to be disease modifying” http://www.prweb.com/releases/2014/1/prweb11489644.htm. Although these findings have not yet been peer-reviewed, if confirmed they provide exciting preclinical data that may be used to formulate human clinical trials in the near future.

The story of caspase-cleavage of tau in AD represents how studying the basic mechanisms underlying a disease can lead to a better understanding of the disease process as well as identifying new drug targets. As an investigator who has spent his entire academic career studying the role of caspases in neurodegenerative diseases, it is gratifying to see the realization of such research from the bench to the clinic.

## Figures and Tables

**Figure 1:**
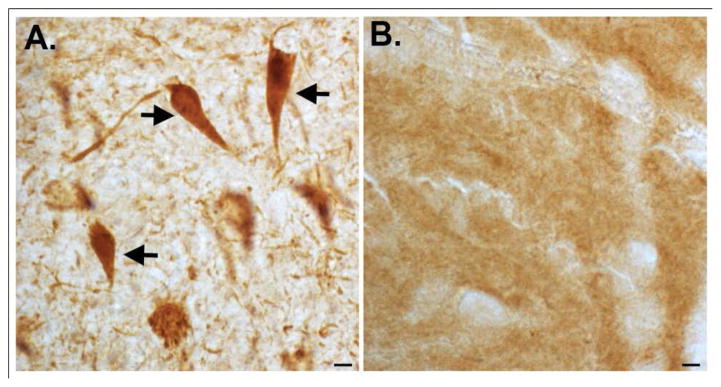
First known demonstration of the caspase-cleavage of tau in the human AD brain. On October 21, 2001 we performed an immuno-histochemical experiment on hippocampal brain sections utilizing a purified caspase-cleavage antibody to tau. The results indicated strong labeling in NFTs of the AD brain (arrows, A), that were absent in age-matched control sections (B). Scale bars represent 10μm.
